# Kamishoyosan reduces anxiety-like behavior by modulating GABAA receptor subunit expression in a premenstrual syndrome rat model

**DOI:** 10.3389/fpsyt.2026.1799904

**Published:** 2026-10-16

**Authors:** Shiori Harada-Nitta, Keisuke Ogura, Kunihiro Konno, Miwa Nahata, Naoki Fujitsuka, Yohei Tokita

**Affiliations:** 1 TSUMURA Kampo Research Laboratories, TSUMURA & CO., Ibaraki, Japan; 2 TSUMURA Advanced Technology Research Laboratories, TSUMURA & CO., Ibaraki, Japan; 3 Research and Development Division, TSUMURA & CO., Tokyo, Japan

**Keywords:** allopregnanolone, GABAA receptor α4 subunit, Kamishoyosan, premenstrual syndrome, progesterone, progesterone withdrawal

## Abstract

**Introduction:**

Premenstrual syndrome (PMS) and premenstrual dysphoric disorder (PMDD) involve psychological symptoms that impact women’s quality of life. PMS pathogenesis is characterized by fluctuations in steroid hormones and γ-aminobutyric acid type A receptor (GABAAR) subunits. Kamishoyosan (KSS) is a Kampo medicine typically used to treat menstrual-related symptoms. This study aimed to elucidate the anxiolytic effect of KSS and its underlying mechanisms in a progesterone withdrawal (PWD)-induced PMS rat model.

**Methods:**

KSS was administered via feed mixing, and elevated plus maze test and molecular biological analysis were conducted.

**Results:**

PWD rats showed increased anxiety-like behavior and expression of GABAAR α4 and δ subunits mRNA in the hippocampal CA1 region, suggesting that decreased inhibitory GABA synaptic signaling contributes to anxiety-like behavior. KSS administration reduced anxiety-like behavior and the expression of GABAAR α4 and δ subunits, but no significant effect was observed on steroid hormone levels.

**Discussion:**

These findings suggest that KSS exerts anxiolytic effects by reversing changes in α4 and δ subunit expression without affecting steroid hormone fluctuations. The different mechanism of action of KSS compared with GABAAR agonists may provide a novel therapeutic strategy for PMS.

## 1 Introduction

Premenstrual syndrome (PMS) is a common condition experienced by women of reproductive age during the luteal phase of the menstrual cycle and characterized by physical, behavioral, and mood symptoms. A meta-analysis revealed varying PMS prevalence by country; in addition, while PMS symptoms and severity differ among individuals, >90% of women aged 20–49 experience them (1). According to the Diagnostic and Statistical Manual of Mental Disorders (2), when ≥5 symptoms are present—at least one psychological and one physical symptom—the condition is diagnosed as premenstrual dysphoric disorder (PMDD) (3). PMDD not only severely impacts daily life, academic performance, employment, and interpersonal relationships but has also been linked to an increased risk of perinatal depression (4, 5), requiring appropriate treatment.

While serotonin is associated with the psychological symptoms of PMS and PMDD, recent research has revealed that hormonal balance changes, particularly progesterone fluctuations, are implicated in PMS pathogenesis (6). Progesterone levels increase during the luteal phase and decrease just before menstruation. Its metabolite, allopregnanolone (ALLO), shows a similar pattern (7). ALLO acts as a potent allosteric modulator of γ-aminobutyric acid A receptors (GABA_A_R) in the brain (8), exerting anxiolytic, anti-stress, and antidepressant effects by enhancing GABA action (9). The current prevailing view is that fluctuations in ALLO levels and impaired GABA_A_R responses contribute to the pathogenesis of PMS (10). In PMS model animals, abrupt progesterone withdrawal (PWD) induces anxiety- and depression-like behavior (11–13). Further, in several brain regions—including the hippocampus, amygdala, and periaqueductal gray matter (PAG)—expression of GABA_A_R α4 and δ subunits increases, an alteration thought to contribute to the behavioral changes observed in PWD rats (14).

In clinical practice, several therapeutic strategies are used to manage PMS symptoms. Current treatment strategies include both pharmacological and nonpharmacological approaches. According to the guidelines of the American College of Obstetricians and Gynecologists (15), selective serotonin reuptake inhibitors are recommended as first-line pharmacological treatment for moderate–severe symptoms, particularly affective symptoms, while hormonal therapies, such as combined oral contraceptives, are used to regulate ovarian hormone fluctuations. In addition, nonpharmacological interventions, including lifestyle modification, such as regular exercise, dietary adjustments, stress management, and cognitive behavioral therapy, may help alleviate symptoms in some patients. However, these treatments do not exhibit uniform efficacy and may be associated with adverse effects, highlighting the need for additional therapeutic options.

Recently, herbal medicines have received attention as complementary therapies for gynecological disorders. Kamishoyosan (KSS) is a traditional Japanese medicine (Kampo) widely used to treat menstrual irregularities, dysmenorrhea, menopausal disorders, as well as women’s mental and physical ailments associated with hormonal fluctuations. It is frequently prescribed for PMS after OC treatment (16). Previously, KSS was reported to improve symptoms in patients with PMS whose primary complaints were mental symptoms such as irritability and headaches (17) Further, Iba-Tanaka et al. reported that KSS attenuates anxiety-like behavior in PMS model rats by increasing brain serotonin levels and activating serotonin 1A receptors (18) In contrast, Mizowaki et al. reported that a 5α-reductase inhibitor and a GABA_A_R antagonist blocked the anxiolytic effect of KSS in male mice (19), suggesting that KSS may alleviate psychological distress through neurosteroid synthesis and GABA_A_Rs signaling. In general, 5α-reductase is involved in testosterone metabolism but also participates in progesterone metabolism, suggesting its involvement in the anxiolytic effect of KSS in female animals. While evidence supporting its therapeutic effects on anxiety is increasing, the effects of KSS on neurosteroid levels and GABA_A_Rs in PMS remain unclear.

Therefore, this study aimed to investigate the mechanism of action of KSS on the psychological symptoms of PMS. Further, we examined the involvement of GABA_A_Rs and sex steroid hormones in the anxiolytic effects of KSS in PWD rats.

## 2 Materials and methods

### 2.1 Animals

Female Wistar rats (9 weeks old) were purchased from Japan SLC, Inc. (Shizuoka, Japan). Rats were housed in groups of 2–3 per cage under a 12/12 h light-dark cycle at a constant temperature (23 ± 3°C) and humidity (50 ± 20%). Rats had free access to food (MF diet; Oriental Yeast, Tokyo, Japan) and water. All animal experiments were conducted in-house at the animal facility of TSUMURA & CO., accredited by the Japan Pharmaceutical Information Center. The experiments were approved by the institutional animal care and use committee of TSUMURA & CO. (approval no.23-021). We confirm that all studies were performed in accordance with the ARRIVE guidelines.

### 2.2 Determination of the estrous cycle phase

After collecting vaginal smears using a swab (4.7 mm diameter) moistened with distilled water, the smears were applied to glass slides. For routine estrous cycle monitoring, sampling was performed daily between 9:00 and 10:00 a.m. For the exploratory behavioral analysis, vaginal smears were collected immediately after the elevated plus-maze (EPM) test. Following air-drying, the slides were fixed with methanol, stained with Giemsa’s solution (FUJIFILM Wako, Osaka, Japan), and observed under a microscope BH-2 BHS (OLYNPUS, Tokyo, Japan). Cycles were classified into seven stages (P, PE, E, EM, M, MD, and D) based on a previous study (20). For evaluation of the estrous cycle, these stages were grouped into three phases: (1) non-estrus (D, MD, M, EM), (2) estrus (E), and (3) proestrus (PE, P). To examine whether progesterone administration affects the estrous cycle, the occurrence rate of each phase was calculated for two periods: 4 days before progesterone administration and 6 days thereafter (Days 2–7). For the exploratory behavioral analysis, animals were categorized as either non-estrus (D, MD, M, EM) or estrus (E, PE, and P) because the number of animals in each individual phase was limited. The association between estrous cycle phase and EPM outcomes was then evaluated.

### 2.3 Drugs and reagents

Dried powdered KSS extracts (lot no. 2230024010 and 2240024010) were supplied by TSUMURA & CO. (Tokyo, Japan) and manufactured as follows: the mixture of the constituent crude drugs (**Supplementary Table S1**) was extracted with purified water at 95°C for 1 h. Then, the extraction solution was separated from the insoluble waste, concentrated by water removal under reduced pressure, and spray-dried to produce a dried extract powder. **Supplementary Figure S1** shows the representative ingredients of KSS identified by a three-dimensional HPLC profile provided by TSUMURA & CO. An MF diet containing 3% (w/w) KSS was prepared by Oriental Yeast (Tokyo, Japan), and progesterone was purchased from FUJIFILM Wako Pure Chemical Corporation (Osaka, Japan). The dietary KSS concentration corresponded to an estimated dose of ~2,000 mg/kg/day. Sesame oil was purchased from Sigma-Aldrich Co. (St. Louis, MO, USA).

PWD was performed with some modifications in the method previously reported (21). Briefly, progesterone (12 mg/0.2 mL sesame oil/rat) was administered intraperitoneally once daily for six days between 8:30 and 11:30 AM, followed by a 24-h drug-free period. Intraperitoneal injection was chosen because progesterone administered via this route is rapidly absorbed into the systemic circulation, as previously reported (22), allowing a rapid decline in blood progesterone levels after withdrawal. After withdrawal, rats were either behaviorally tested or sacrificed by exsanguination under deep anesthesia to collect blood and brain samples.

### 2.4 Experimental design

The experimental timeline is depicted in **Figure 1**. A total of 226 rats were assigned to one of the following two or three groups based on their weight at the start of the experiment: Control group (vehicle injection + MF diet), PWD group (PWD + MF diet), and PWD + KSS group (PWD + 3%-KSS diet). PWD + KSS rats were fed a 3% KSS diet from four days before progesterone injection until the end of the experiment. Both control and PWD rats received the MF diet. Sample sizes differed among experiments because of differences in experimental design and technical considerations. In Experiments 2 and 5, two animal batches were used to perform two comparisons: (1) Control vs. PWD and (2) PWD vs. PWD + KSS.

**Figure 1:**
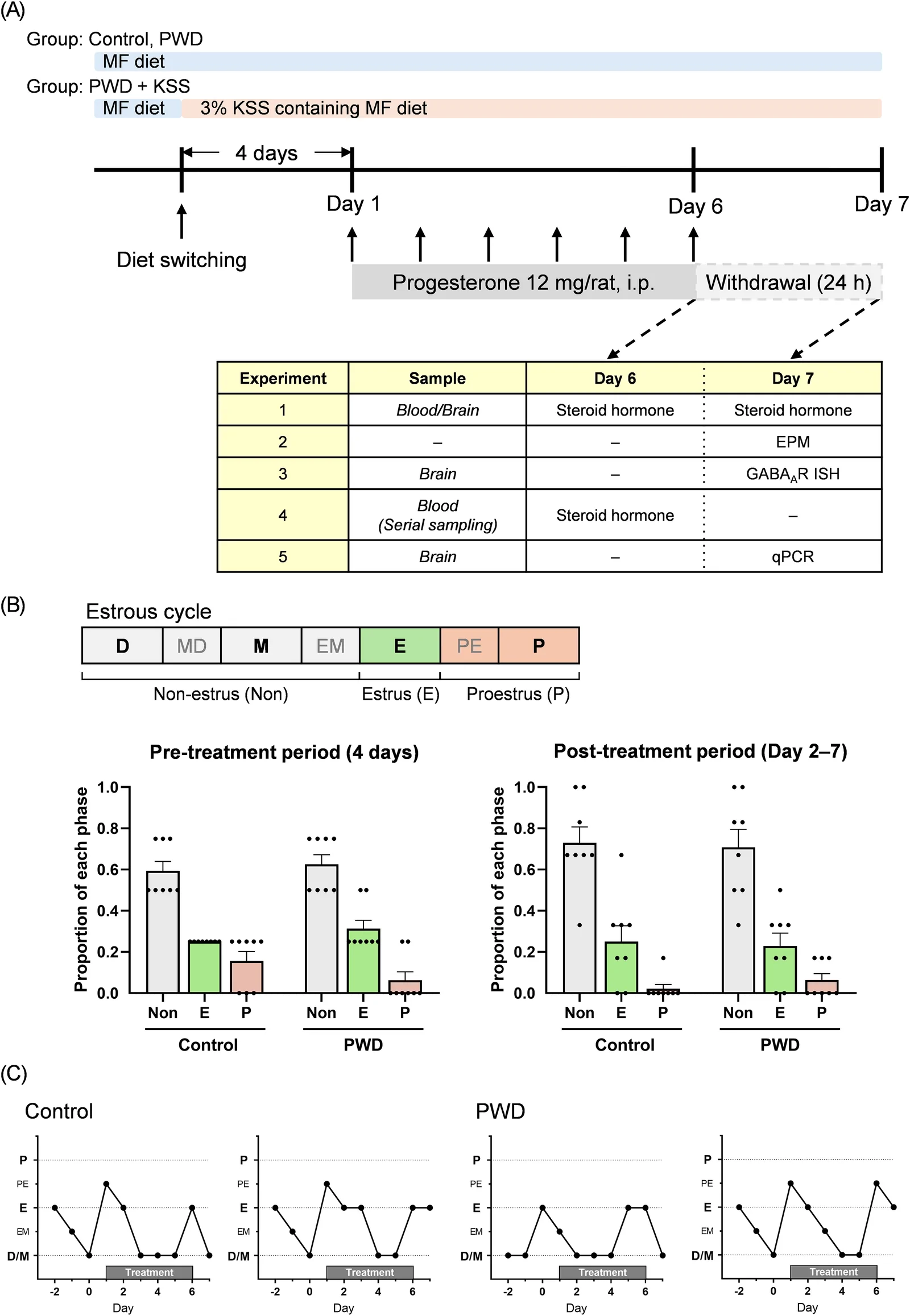
Experimental timeline and estrous cycle. **(A)** Rats were intraperitoneally injected with 12 mg/rat progesterone once a day for six days and subjected to 24 h of withdrawal. In each experiment, rats were either subjected to behavioral tests or sacrificed to collect blood or brain samples at the timepoint indicated in the scheme. Progesterone withdrawal (PWD) + KSS rats received a 3% kamishoyosan (KSS) containing MF diet from four days before the beginning of progesterone injections until the end of the experiment. Both Control and PWD rats received an MF diet. **(B)** Proportion of days in each estrous cycle phase. Phases were classified as non-estrus (D/MD/M/EM), estrus (E), and proestrus (PE/P). Proportions were calculated for the 4 days before and 6 days after progesterone administration (Days 2–7). Proportions represent compositional data (Non + E + P = 1 for each individual); n = 8. **(C)** Representative estrous cycle patterns in control and PWD rats before and after progesterone administration. The gray bar indicates the period of progesterone or vehicle administration. EPM, elevated plus maze; ISH, *in situ* hybridization; qPCR, quantitative real-time polymerase chain reaction; D, diestrus; M, metestrus; E, estrus; P, proestrus; MD, metestrus–diestrus; EM, estrus–metestrus; PE, proestrus–estrus.

#### 2.4.1 Experiment 1

To confirm fluctuations in steroid hormones in PWD rats, the brain and blood samples of control and PWD rats were collected under deep anesthesia with sevoflurane 1 h after the last progesterone injection (Day 6, n = 8) and after withdrawal (Day 7, n = 8). After collecting the whole brain, the hippocampus was dissected and stored at −80°C until analysis. Blood was collected from the abdominal vena cava using a heparinized syringe and needle. Then, the samples were centrifuged (3,300 rpm, 5 minutes, 4°C) to separate the plasma, which was then stored at −80°C until analysis. Brain and plasma samples were used for steroid hormone measurement by liquid chromatography-mass spectrometry (LC-MS/MS).

#### 2.4.2 Experiment 2

The EPM test for anxiety assessment was conducted on Day 7. To minimize circadian rhythm effects, the number of animals tested per day was limited, and two batches of animals were used in two separate sessions: (1) control group vs. PWD group (n = 7), and (2) PWD group vs. PWD + KSS group (n = 7). To examine the relationship between estrous cycle phase and EPM performance, an additional cohort of control (n = 27) and PWD rats (n = 24) underwent estrous cycle phase assessment immediately after EPM testing.

#### 2.4.3 Experiment 3

To confirm the distribution of GABA_A_R subtypes, the brains of control rats (n = 12), PWD rats (n = 12), and PWD + KSS rats (n = 12) were collected under deep anesthesia with sevoflurane after PWD (Day 7). Brain samples were processed as described below and *in situ* hybridization performed in the hippocampal CA1 region. A larger number of animals per group was used because the evaluation was performed in a very limited brain region.

#### 2.4.4 Experiment 4

To confirm the effect of KSS on steroid hormone fluctuations in PWD rats, the brain and plasma of PWD rats (n = 3–4) and PWD + KSS rats (n = 4) were collected under deep anesthesia with sevoflurane before and at 30, 60, 120, and 240 min after the final progesterone injection (Day 6). Both the hippocampus and plasma samples were used for steroid hormone measurement by LC-MS/MS.

#### 2.4.5 Experiment 5

To perform mRNA expression analysis of GABA signaling-regulating molecules, brain samples were collected under deep anesthesia with sevoflurane after progesterone withdrawal (Day 7). The hippocampal CA1 region was microdissected and used for mRNA expression analysis by quantitative real-time polymerase chain reaction (qPCR). To minimize the technical variability associated with the collection of specific and limited brain regions, the number of animals and groups was adjusted, and the experiment conducted in two separate sets: (1) control group vs. PWD group (n = 8), and (2) PWD group vs. PWD + KSS group (n = 12).

### 2.5 Elevated plus-maze test

The EPM test is a behavioral experiment primarily used to evaluate anxiety in animals. The EPM apparatus (Shinfactory, Fukuoka, Japan) used in this test was cross-shaped and comprised a central platform (10 ×10 cm) and four arms (length 50 cm × width 10 cm). The two arms (50 × 10 cm) were open and faced each other. The remaining two arms are enclosed by walls (40 cm high). The maze was positioned 50 cm above the floor. This design aimed to induce anxiety in animals by placing them in an elevated position. Illumination was set to 5.0 lux in the central part of the maze. Each animal was placed on the central platform with their head toward the open arm; animals’ behavior (movement and trajectory) was recorded for 5 min. The number of entries into the open and enclosed arms and the time spent in each arm type were analyzed using ANY-maze (Stoelting Co., USA). In the EPM test, anxiety levels were assessed by observing the type of arm (open or enclosed) chosen by the animal. Since rats prefer enclosed spaces to open ones, spending more time in the open arms or entering them more frequently were considered to exhibit less anxiety-like behavior. The variables analyzed included % open arm time, anxiety index, the number of open-arm entries, and % enclosed arm time. Variables were calculated and evaluated using the following formula (23).


%open arm time=(time spent in open arms/300)∗100

%enclosed arm time=(time spent in enclosed arms/300)∗100

Anxiety index=1−(((time spent in open arm/300)+(number of open arm entries/total entries))/2)

The number of entries in the closed arms was considered an index of locomotor activity (24). To minimize circadian and day-to-day variability, the EPM test was conducted during the light phase between 09:00 and 14:00 in two batches (control vs. PWD and PWD vs. PWD + KSS). Within each batch, animals were tested in a counterbalanced order. In the exploratory experiment examining the relationship between EPM outcomes and estrous cycle phase, vaginal smears were collected immediately after behavioral testing and classified as described above.

### 2.6 Determination of plasma and brain steroid hormone levels

To minimize diurnal variation, blood samples were collected between 09:00 and 12:00, with animals from each group sampled in an alternating order. All animals belonged to a single experimental batch. Plasma and brain progesterone and ALLO concentrations were determined using LC-MS/MS after sample preprocessing. Plasma samples were first mixed with 4-fold volume (v/v) of methanol, and brain tissues were homogenized and adjusted to a concentration of 70 mg wet weight per mL of ethanol. After treating plasma and brain samples, all samples were centrifuged, and the supernatants mixed with allopregnanolone-2,2,3,4,4-d_5_ (internal standard) and preprocessed by solid-phase extraction using Phree Phospholipid Removal Tubes (Phenomenex Inc., Torrance, CA). Then, the resultant extracts were injected into the LC-MS/MS system for analysis of the two hormones. The analytical system consisted of a Nexera X2 LC system coupled to an LCMS-8060 mass spectrometer (Shimadzu, Kyoto, Japan) equipped with an electron ionization source. Chromatographic separation was performed on a Poroshell EC 120 C18 column (50 × 2.1 mm I.D., 2.7 μm particle size; Agilent Technologies, Inc., Santa Clara, CA) at 40°C. The mobile phase consisted of solution A (0.2% formic acid aqueous solution, v/v) and solution B (methanol), with the following gradient: 10% B (0–1 min), linear increase to 95% B (1–25 min), and re-equilibration to 10% B (25.1–30 min) at 0.4 mL/min. Each analyte was detected under the selected reaction monitoring mode under the following transitions (m/z, Q1/Q3): 319.05/257.25 for allopregnanolone, 315.05/97.10 for progesterone, and 324.05/262.25 for progesterone. allopregnanolone-2,2,3,4,4-d_5_. Data were analyzed using peak analysis software (Cascade; Reifycs Inc., Tokyo, Japan), and each hormone concentration was calculated using a calibration curve based on the relative peak intensity of each hormone to the internal standard. Pharmacokinetic parameters were calculated using Phoenix WinNonlin software version 8.4 (Certara L.P., St. Louis, MO, USA).

### 2.7 *In situ* hybridization

The rat brain tissue was perfusion-fixed with 4% paraformaldehyde (PFA) and postfixed in a 4% PFA solution for 24 h at room temperature. Formalin-fixed paraffin-embedded block samples were prepared by paraffin replacement of brain tissue using Tissue-Tec VIP 5 Jr. (Sakura Finetek Japan Co., Ltd., Tokyo, Japan) and paraffin embedding using Tissue-Tec TEC (Sakura Finetek Japan Co., Ltd., Tokyo, Japan). *In situ* hybridization was performed by Morpho Technology (Hokkaido, Japan) using RNAscope™ Multiplex Fluorescent Reagent Kit v2 (Advanced Cell Diagnostics, Hayward, CA) with standard protocols. Rn-Gabra4-C1 and Rn-Gabrd-C1 probes were provided by Advanced Cell Diagnostics (Hayward, CA, catalog #410771 and 1073711-C1, respectively). Hybridization products for Gabra4 and Gabrd were visualized using OPAL 570 (1:750 dilution, Akoya Biosciences, Marlborough, MA, USA). Using the stained images generated, we quantified gene expression by counting labeled probes in-house using the trainable Weka segmentation plugin for ImageJ (US National Institutes of Health, Bethesda, MD, USA) and quantified gene expression.

### 2.8 Gene expression

Rat brains were sectioned into 1 mm thick coronal sections using a rat brain matrix (ASI Instruments, Warren, MI), and tissue samples from the CA1 region of the hippocampus in each hemisphere were excised using a punch (BrainScience idea, Osaka, Japan) to obtain two samples with a diameter of 1 mm each. A total of four tissue samples per animal were pooled and homogenized using QIAzol (Qiagen, Venlo, Netherlands), total RNA was extracted using the RNeasy Mini kit (Qiagen), and reverse transcription reaction was performed using the TaqMan High-Capacity Reverse Transcription Kit (Thermo Fisher Scientific, Waltham, MA, USA). qPCR assays were performed using TaqMan Fast Advanced Master Mix (Thermo Fisher Scientific) and the QuantStudio 5 Real-Time PCR System (Thermo Fisher Scientific). Each mRNA expression was normalized to that of the endogenous control β-actin (Actb). TaqMan gene-specific primer-probe sets were Actb, Rn00667869_m1; glutamate decarboxylase 1 (GAD1/GAD67), Rn00690300_m1; glutamate decarboxylase 2 (GAD2/GAD65), Rn00561244_m1; solute carrier family 32, member 1 (vesicular GABA transporter; VGAT), Rn00824654_m1; solute carrier family 6, member 1 (GABA Transporter 1; GAT-1), Rn00577652_m1; early growth response 3 (EGR3), Rn00567228_m1.

### 2.9 Statistical analysis

Statistics were calculated using Prism 7 (GraphPad Software, San Diego, CA, USA). Data are shown as mean ± SEM. For testing homogeneity of variance, the F-test was used for comparisons between two groups, and Bartlett’s test for comparisons involving ≥3 groups. Based on the results of these tests, the subsequent analyses described below were employed. To evaluate hormone levels in PWD rats, Kruskal–Wallis test followed by Dunn’s multiple comparisons test was used. For the EPM test, original comparisons within each experimental batch were performed using an unpaired *t*-test. Data from the combined dataset were adjusted for batch effects associated with the experimental date using a linear model in R software (version 4.3.1; R Foundation for Statistical Computing, Vienna, Austria, https://www.R-project.org/). The adjusted data were subsequently analyzed using a Kruskal–Wallis test followed by Dunn’s multiple-comparison test. To evaluate the potential influence of the estrous cycle on EPM performance, control and PWD rat data were additionally analyzed by two-way analysis of variance (ANOVA), with the treatment group and estrous cycle phase as factors. The quantification data of GABA_A_R subtypes were analyzed using one-way ANOVA followed by Tukey’s multiple comparison test. Steroid hormone fluctuations were analyzed using Tukey’s multiple comparison test after one-way ANOVA, or Dunn’s test (for unequal variances) after the Kruskal–Wallis test. For gene expression analysis experiments for GABA signaling molecules, the original comparisons within each experimental batch were performed using an unpaired *t*-test. Data from the combined dataset were adjusted for batch effects associated with the experimental date using a linear model in R. The adjusted data were subsequently analyzed using one-way ANOVA, followed by Tukey’s multiple-comparison test. *P* values< 0.05 (two-tailed) were considered statistically significant. Power analysis based on the observed effect sizes (Cohen’s d) of the behavioral data was conducted using R software.

## 3 Results

### 3.1 Estrous cycle phase

**Figure 1A** shows the experimental schedule. Comparisons of body weight between groups at the start and end of the experiment revealed no significant differences among the control, PWD, and PWD + KSS groups at either time point (start: control 158.4 ± 2.0 g, PWD 158.7 ± 2.1 g, PWD + KSS 157.4 ± 2.5 g; end: control 176.5 ± 2.4 g, PWD 181.7 ± 2.9 g, PWD + KSS 177.1 ± 3.0 g; mean ± SEM). First, we examined whether progesterone administration affects the estrous cycle. We calculated the proportion of estrous cycle phases occurring during two independent periods: the 4 days before progesterone administration and the 6 days thereafter (Days 2–7) (**Figure 1B**). No difference in the proportion of estrous cycle phases was observed between the control and PWD groups during either period. Although the variation in this proportion increased in the second period compared to the first, a similar increase in variation was observed in the control group. Representative estrous cycles are shown in **Figure 1C**.

### 3.2 Hormone levels in PWD rats

As shown in **Figure 1A**, progesterone was administered intraperitoneally for six days, followed by a 24-h withdrawal period. Brain and blood samples were collected 1 h after the final progesterone injection (Day 6) and after progesterone withdrawal (Day 7) to measure progesterone and ALLO concentrations. The results are shown in **Table 1**. Compared with control rats, PWD rats exhibited increased progesterone levels in plasma and hippocampus on Day 6, declining to levels comparable to the control group on Day 7. Plasma and hippocampal ALLO levels showed a similar pattern to progesterone. Kruskal–Wallis test results were as follows: plasma progesterone (H = 13.74, df = 3, *p* = 0.0033), hippocampal progesterone (H = 6.645, df = 3, *p* = 0.0841), plasma ALLO (H = 18.79, df = 3, *p* = 0.0003), and hippocampal ALLO (H = 18.82, df = 3, *p* = 0.0003).

**Table 1:** Progesterone and allopregnanolone levels in plasma and the hippocampus.

Analyte	Sample	Control		PWD	
		Day 6	Day 7	Day 6	Day 7
Progesterone	Plasma (ng/mL)	8.54 ± 1.35	39.04 ± 13.59	1548.59 ± 600.99 ^*^	8.09 ± 1.88 ^##^
	Hippocampus (ng/g tissue)	53.42 ± 40.83	41.66 ± 16.85	461.26 ± 135.64	55.03 ± 18.98
Allopregnanolone	Plasma (ng/mL)	4.70 ± 1.26	5.76 ± 0.79	277.65 ± 79.02 ^**^	4.29 ± 0.80 ^##^
	Hippocampus (ng/g tissue)	4.51 ± 0.89	4.43 ± 0.40	446.51 ± 125.61 ^***^	6.75 ± 1.37 ^#^

Data are presented as mean ± SEM (n = 8). Since the assumption of homogeneity of variance was not satisfied, data were analyzed using the Kruskal–Wallis test followed by Dunn’s multiple comparisons test for all pairs of groups. Asterisks indicate significant differences between the control and progesterone withdrawal (PWD) groups on the same day (*p < 0.05, **p < 0.01, ***p < 0.001). Hash marks indicate significant differences between Days 6 and 7 within the same group (^#^*p* < 0.05, ^##^*p* < 0.01). No significant differences were observed between control (Day 6) and control (Day 7), or between control (Day 7) and PWD (Day 7).

### 3.3 Anxiety-like behavior in the elevated plus-maze test

**Figure 2A** shows typical behavioral trajectories for each group in the EPM test. In the original batch-specific comparison, PWD rats exhibited anxiety-like behavior characterized by reduced open-arm exploration, higher anxiety index, fewer open-arm entries, and increased enclosed arm time compared with control rats in batch 1 (**Supplementary Figures S2A–D**). In batch 2, KSS administration attenuated these behavioral alterations (**Supplementary Figures S2E–H**). In addition, no differences were observed in the number of enclosed arm entries between groups in either batch 1 (control, 9.29 ± 1.41; PWD, 9.29 ± 1.19; *p* > 0.9999) or batch 2 (PWD, 9.71 ± 1.27; PWD + KSS, 9.57 ± 0.97; *p* = 0.9302), suggesting that locomotor activity was not affected. Despite the limited sample size, effect sizes were consistently large across behavioral measures (Hedges’ *g* = 1.02–1.71), supporting the robustness of the observed effects, although observed power varied among indices (0.42–0.83). To evaluate anxiety-like behavior across all animals while accounting for inter-batch variability, data from the two experimental sets were combined after adjustment for potential batch effects and analyzed as a three-group comparison (**Figures 2B–E**). This analysis confirmed the anxiety-like phenotype observed in the original comparison and enabled evaluation of KSS treatment effects in a unified three-group framework. PWD rats spent significantly less time in the open arms than control rats, whereas KSS-treated rats showed a tendency toward recovery (**Figure 2B**; H = 9.859, df = 2, *p* = 0.0072). Similarly, PWD rats had a higher anxiety index than control rats, and KSS-treated rats showed a tendency toward recovery (**Figure 2C**; H = 11.84, df = 2, *p* = 0.0027). The number of open-arm entries was significantly lower in PWD rats and increased significantly by KSS treatment (**Figure 2D**; H = 15.09, df = 2, *p* = 0.0005). In contrast, the percentage of time spent in the enclosed arms was significantly higher in PWD rats than in control rats, with no significant difference between the PWD and PWD + KSS groups (**Figure 2E**; H = 11.82, df = 2, *p* = 0.0027).

**Figure 2:**
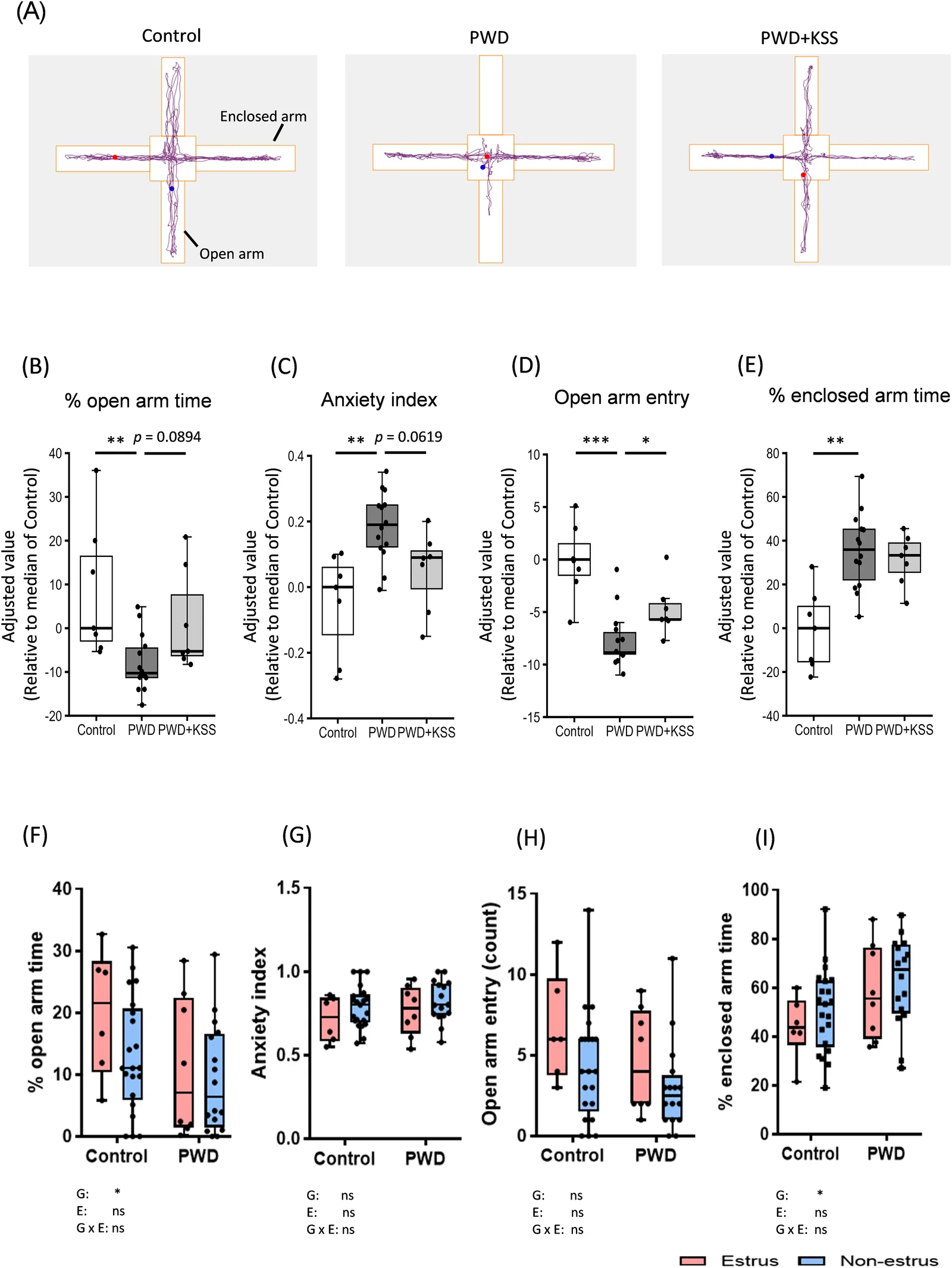
Batch-adjusted three-group comparison of anxiety-like behavior in the elevated plus maze. **(A–E)** Behavioral experiments were conducted in two separate batches (batch 1: Control vs. PWD; batch 2: PWD vs. PWD + KSS; n = 7 per group). Representative trajectory diagram **(A)** showing the path of the animal’s center point. The blue dot indicates the start of the trajectory, and the red dot indicates the end. **(B)** Percentage of time spent in the open arms (% open arm time), **(C)** anxiety index, **(D)** number of open-arm entries, and **(E)** percentage of time spent in the enclosed arms (% enclosed arm time). Data from the two experimental sets were combined after adjustment for potential batch effects using a linear model. Group differences were analyzed using the Kruskal–Wallis test, followed by Dunn’s multiple-comparison test. Statistically significant differences are indicated as *p < 0.05, **p < 0.01, ***p < 0.001. **(F–I)** Analyses stratified by group (Control, n = 27; PWD, n = 24) and estrous cycle phase (estrus, including the estrus and proestrus stages, vs. non-estrus). Results of the two-way ANOVA are shown in each panel. The main effects of group and estrous cycle phase, denoted as G and E in the ANOVA labels, respectively, and their interaction (G × E) are indicated by asterisks (*) for statistically significant effects and “ns” for nonsignificant effects. Data are presented as box-and-whisker plots showing the median, interquartile range, and minimum-to-maximum values.

To determine whether the estrous cycle phase influenced EPM performance, an additional cohort of control and PWD rats was analyzed according to treatment group and estrous cycle phase. As PWD did not alter estrous cycle distribution compared with controls (**Figures 1B, C**), the potential contribution of the estrous cycle phase to EPM performance was assessed using these two groups. The PWD + KSS group was not included in this exploratory analysis because the primary aim was to determine whether the behavioral phenotype induced by PWD was influenced by the estrous cycle phase. The control group included six rats in estrus and 21 in non-estrus phases, whereas the PWD group included eight rats in estrus and 16 in non-estrus phases. Two-way ANOVA revealed no significant interaction between treatment group and estrous cycle phase for any EPM parameter (**Figures 2F–I**). Likewise, no significant main effect of estrous cycle phase was detected for any parameter. In contrast, a significant main effect of treatment group was observed for the percentage of time spent in the open arms (% open arm time; *F*(1, 47) = 4.309, *p* = 0.0434) and the percentage of time spent in the enclosed arms (% enclosed arm time; *F*(1, 47) = 5.617, *p* = 0.0219). However, Turkey’s *post hoc* multiple-comparison test did not identify significant differences among individual subgroups (**Figures 2J–M**). These findings indicate that the differences in EPM performance were primarily attributable to PWD exposure rather than the estrous cycle phase.

### 3.4 Distribution of GABA_A_R subtypes in the hippocampus

*In situ* hybridization assays showed increased GABA_A_R α4 subunit expression in pyramidal cells of the hippocampal CA1 region with PWD (**Figures 3A** top). In PWD rats, an increase in the α4 subunit was also observed in the outer layers of the pyramidal cell layer (**Figures 3A** bottom). The δ subunit was similarly expressed in pyramidal cells and upregulated by PWD (**Figure 3B**). Conversely, the increased expression of both subunits was suppressed by KSS (**Figures 3A, B**). The increased expression levels of both subunits due to PWD and the suppression induced by KSS administration were significant when counting the number of label probes per cell (**Figures 3C, D**). One-way ANOVA revealed significant group differences (α4: *F*(2, 33) = 16.96, *p* < 0.0001; δ: *F*(2, 33) = 11.46, *p* = 0.0002).

**Figure 3:**
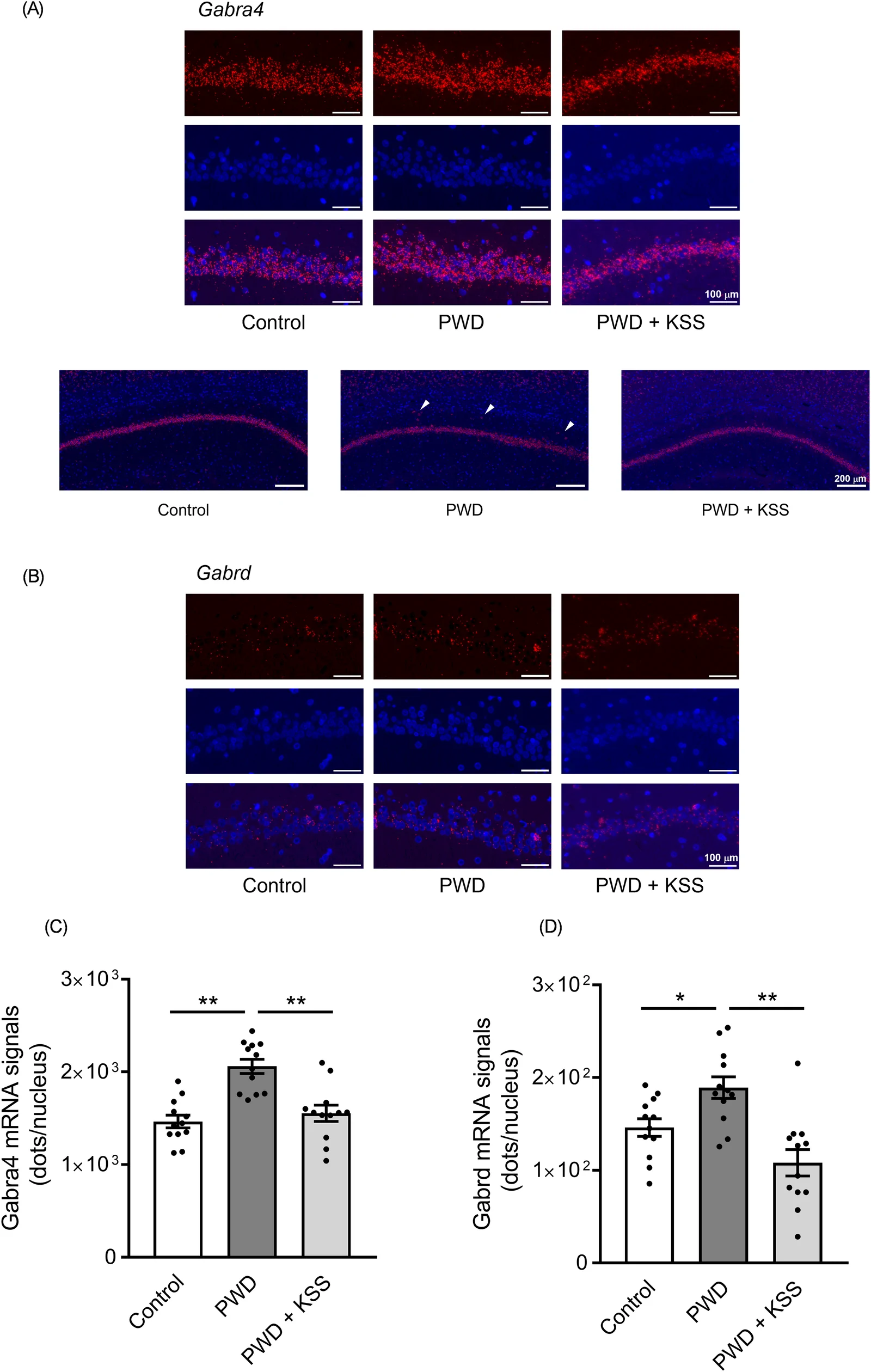
GABA_A_ receptor subunit expression in the hippocampus. **(A, B)** Representative fluorescence *in situ* hybridization images showing Gabra4 **(A)** and Gabrd **(B)** mRNA expression in the hippocampal CA1 region. For each condition in the nine panels, the top row shows the mRNA signal (red), the central row cell nuclei counterstained with DAPI (blue), and the bottom row shows the merged image. Scale bars, 100 µm. The three large panels are low-magnification images of the merged Gabra4. Scale bars, 200 µm. **(C, D)** Quantification of Gabra4 **(C)** and Gabrad **(D)** mRNA expression (n = 12). Each data represents the mean ± SEM. Statistical analysis was performed using Tukey’s test. Statistically significant differences are indicated as *p < 0.05, **p < 0.01. PWD, progesterone withdrawal; KSS, kamishoyosan.

### 3.5 Steroid hormone fluctuation after progesterone injection

To verify whether differences in ALLO levels were responsible for the observed changes in GABA_A_R subunit composition, we measured fluctuations in both progesterone and ALLO levels in the plasma and hippocampus immediately after the final progesterone injection on Day 6 for 240 min. Before the injection, there was no difference between the PWD and PWD + KSS groups. After progesterone injection, plasma and hippocampal levels peaked at 30 or 60 min and declined at 120 or 240 min (**Figures 4A–D**, **Table 2**). No obvious difference was observed in the pharmacokinetic parameters between the groups (**Table 2**).

**Figure 4:**
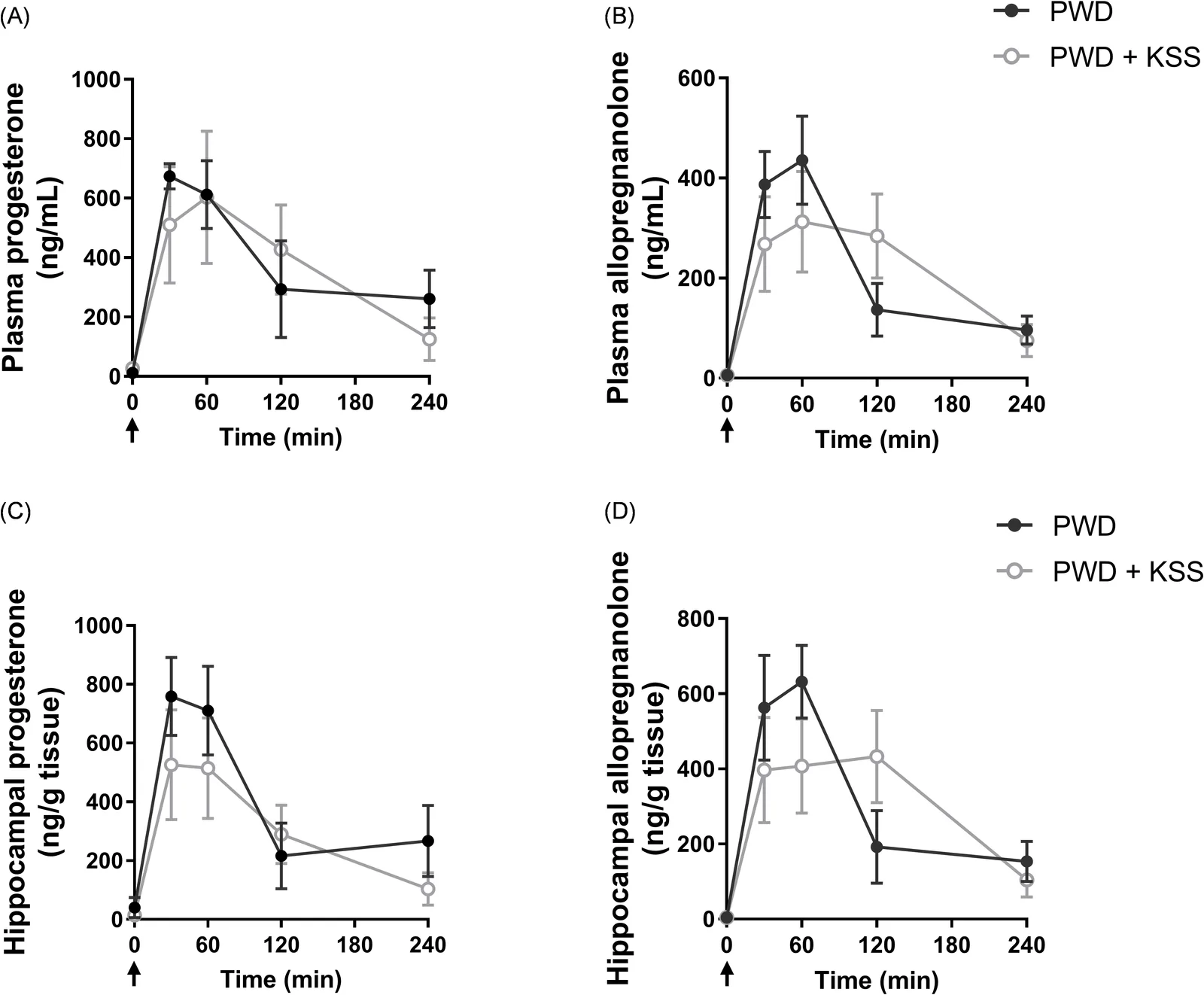
Fluctuation of peripheral steroid hormones after progesterone treatment. Progesterone and allopregnanolone levels in the plasma **(A, B)** and hippocampus **(C, D)** after intraperitoneal progesterone injection on Day 6. The arrow indicates the time of progesterone administration. Each data represents the mean ± SEM (n = 3–4). PWD, progesterone withdrawal; KSS, kamishoyosan.

**Table 2:** Pharmacokinetic parameters of progesterone and allopregnanolone in plasma and the hippocampus.

Pharmacokinetic parameter	Plasma				Hippocampus			
	Progesterone		Allopregnanolone		Progesterone		Allopregnanolone	
	PWD	PWD + KSS	PWD	PWD + KSS	PWD	PWD + KSS	PWD	PWD + KSS
T_max_ (min)	30.00	60.00	60.00	60.00	30.00	30.00	60.00	120.00
C_max_ (ng/mL)	674.00	602.73	435.67	312.70	759.00	525.98	632.33	433.00
AUC_0-240_ (ng min/mL)	88864.93	84904.86	47587.09	49556.42	87924.85	68940.07	69275.40	71022.76

PWD, progesterone withdrawal; KSS, kamishoyosan; Cmax, peak concentration; Tmax, time to peak concentration; AUC, area under the concentration-time curve. All values were calculated using the mean concentration (n = 3–4) per time point.

### 3.6 GABA signaling molecules

Next, we performed gene expression analysis using qPCR to determine the factors regulating GABA and GABA_A_R subunit expression in the hippocampal CA1 region. In the original batch-specific comparison, no significant differences in the mRNA expression of the GABA synthesizing enzymes GAD1/GAD67 and GAD2/GAD65, VGAT, or EGR3 that regulate GABA_A_R subunit expression were observed between the control and PWD groups (**Supplementary Figures S3A–C, E**). In contrast, the expression of GAT-1, responsible for GABA reuptake from the synaptic cleft, was significantly increased in the PWD group compared to the control group (**Supplementary Figure S3D**). KSS administration had no effect on any of these factors (**Supplementary Figures S3F–J**). To account for potential batch effects associated with the experimental date, data from all experimental batches were combined after adjustment for batch effects and analyzed using a three-group comparison (**Figures 5A–E**). In this analysis, no significant differences in the expression of GAD1/GAD67, GAD2/GAD65, VGAT, EGR3, or GAT-1 were detected among the groups. Although the original comparison suggested an increase in GAT-1 expression in PWD rats, this effect was not maintained after adjustment for batch effects and inclusion of all experimental data, indicating that the observed difference was modest and not robust across datasets.

**Figure 5:**
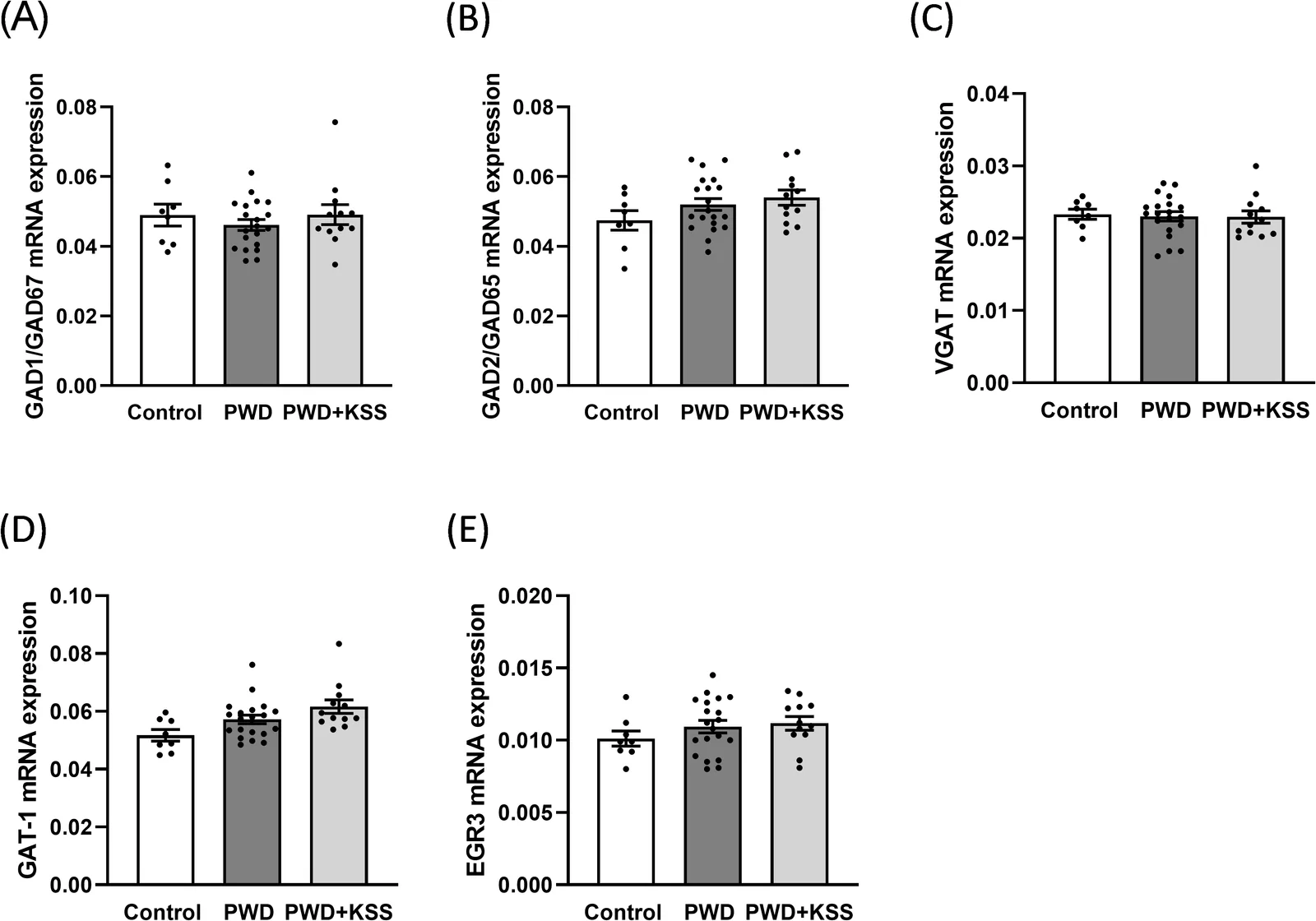
Batch-adjusted three-group comparison of expression of molecules involved in GABA signaling regulation in the hippocampus. **(A–E)** Gene expression analyses were conducted in two separate batches (batch 1, control vs. PWD, n = 8 per group; batch 2, PWD vs. PWD + KSS, n = 12 per group). mRNA expression of molecules involved in GABA synthesis, transport, and reuptake as well as regulator of GABA_A_ receptor α4 subunit expression in the hippocampal CA1 region: GAD1 **(A)**, GAD2 **(B)**, VGAT **(C)**, GAT-1 **(D)**, and EGR3 **(E)**. Data from the two experimental sets were combined after adjustment for potential batch effects using a linear model. Gene expression levels are presented as normalized 2^-ΔCt values. Group differences were analyzed using one-way ANOVA, followed by Tukey’s multiple-comparison test. Data are presented as mean ± SEM. GAD1, glutamate decarboxylase-1 (GAD1/GAD67); GAD2, glutamate decarboxylase-2 (GAD2/GAD65); VGAT, vesicular GABA transporter; GAT-1, GABA transporter 1; EGR3, early growth response 3; PWD, progesterone withdrawal; KSS, kamishoyosan.

## 4 Discussion

This study revealed that KSS ameliorated anxiety-like behavior in PWD rats and suppressed hippocampal GABA_A_R α4 and δ subunits expression. No significant effects were observed in systemic or hippocampal steroid hormone dynamics or GABA signaling molecule expression after KSS treatment, suggesting a direct, plastic change in the GABAergic system. These findings indicate a novel potential therapeutic effect of KSS in PMS.

PWD is a widely used animal model mimicking PMS (25) inducing psychological symptoms, including depressive- and anxiety-like behaviors (26, 27). Saavedra et al. reported that abrupt PWD increased anxiety indices, while a gradual progesterone reduction only caused mild anxiety (28), suggesting that a rapid decrease in progesterone is involved in PMS onset. In this study, we confirmed that after a short-term (6 days) once-daily administration of high-dose (12 mg) progesterone, a 24-h withdrawal period induced anxiety-like behavior in rats.

In the central nervous system, progesterone is converted to ALLO by 5α-reductase and 3α-hydroxysteroid oxidoreductase (9). In patients with PMDD, stabilizing ALLO levels with 5α-reductase inhibitors can decrease mental symptoms, such as irritability, anxiety, and sadness, as well as several physical symptoms (29). These findings suggest that fluctuations in ALLO levels rather than progesterone levels contribute to PMS pathogenesis. In this study, progesterone administration induced dynamic changes and a sharp decrease in both plasma and brain ALLO levels within a short time, suggesting that anxiety-like behavior may be associated with brain ALLO levels. ALLO acts as a potent allosteric modulator of GABA_A_R (8), enhancing GABA’s action to exert anxiolytic, anti-stress, and antidepressant effects (9). However, in light of the observation that ALLO levels did not significantly differ between the control and PWD groups after progesterone withdrawal (Day 7), ALLO levels do not appear to contribute to anxiety-like behavior; rather, impaired GABA_A_R responses caused by abrupt ALLO changes may be responsible.

GABA_A_R is a ligand-gated chloride channel composed of five different combinations of its 19 subunits (α1–α6, β1–β3, γ1–γ3, δ, ϵ, θ, π, ρ1–ρ3); the subunit composition influences the receptor’s function and pharmacological properties (30). Particularly, GABA_A_Rs containing α1, α2, α3, and γ2 subunits are widely distributed at synapses throughout the brain and transmit phasic inhibition, whereas GABA_A_Rs containing α4, α5, α6, and δ subunits are primarily localized extrasynaptically in regions such as the cerebellum, dentate gyrus, and cerebral cortex, transmitting tonic inhibition (31), thought to contribute to reduced excitability in neural networks within the brain. During the menstrual cycle, the expression pattern of GABA_A_R subunits changes, with significant alterations in subunit composition and receptor numbers in response to fluctuations in neurosteroid levels, such as progesterone and ALLO (14). In rats, when progesterone levels decline at late diestrus (diestrus II), expression of α4, β1, and δ subunits increases in PAG neurons. Furthermore, during short-term progesterone or ALLO administration or withdrawal after long-term administration, expression of α4 and δ subunits increases in several brain regions, including the hippocampus, amygdala, and PAG. In the hippocampus, progesterone or ALLO withdrawal increases α4 subunit expression in the CA1 region, decreases GABA currents in the pyramidal cell layer, increases seizure incidence, and enhances anxiety-like symptoms (32). In contrast, studies in δ subunit knockout mice reported no anxiety-like behavior after ALLO withdrawal (33); moreover, finasteride, a neurosteroid synthesis inhibitor, significantly reduced hippocampal δ subunit expression in diestrus mice (34). These findings suggest that changes in α4 and δ subunit expression are associated with anxiety-like behavior. The present study revealed a significant increase in α4 and δ mRNA expression in the hippocampal CA1 region of PWD rats. Interestingly, there was increased α4 subunit expression in the pyramidal cell layer of PWD rats as well as outside layers. This suggests that GABA_A_Rs containing the α4 subunit may at least partially increase in GABAergic interneurons. Normally, GABA signaling from GABAergic interneurons suppresses pyramidal neuron excitability. However, an increase in GABA_A_R containing the α4/δ subunits on interneurons leads to interneuron disinhibition, thereby enhancing the excitability of pyramidal neurons (14). Since we did not verify whether increased expression of the α4 subunit occurred specifically on pyramidal cells or interneurons, this needs to be clarified in future research.

The estrous cycle has been reported to influence anxiety-related behavior and GABAergic mechanisms in female rodents (23, 35, 36). In our preliminary observations, the estrous cycle pattern did not differ between the PWD and control groups. Furthermore, despite the greater variability in the estrous cycle pattern after progesterone administration, similar increases in variability were observed in the control group. This suggests that while stressors, such as administration procedures, may cause some fluctuation in the estrous cycle, PWD may induce behavioral and molecular changes independent of the estrous cycle, at least using the current protocol. To further evaluate the potential contribution of estrous cycle phase to the behavioral phenotype observed in this study, EPM performance was analyzed according to estrous cycle phase. No significant main effect of estrous cycle phase or interaction between estrous cycle phase and treatment group was detected. Although previous studies have reported estrous cycle–dependent changes in anxiety-related behavior in female rodents (23, 36), such effects were not observed under the present experimental conditions.

KSS, a traditional Japanese herbal medicine, is a pharmaceutical product approved for clinical use by Japan’s Ministry of Health, Labor and Welfare. KSS improved symptoms in patients with PMS whose primary complaints were mental symptoms such as irritability and headaches (17). Kimura et al. reported a correlation between KSS’s PMS symptom relief effects and its vascular age improvement effects, suggesting that KSS may have alleviated PMS symptoms by correcting autonomic nervous system imbalance (37). Furthermore, in a double-blind, placebo-controlled, randomized, parallel-group study on menopausal symptoms, the KSS improved scores for excitability and irritability in premenopausal women (38). Therefore, KSS may have therapeutic effects on neuropsychiatric symptoms caused by fluctuations in female hormones associated with the menstrual cycle. Currently, an open-label randomized controlled trial is underway to evaluate its efficacy and safety for PMS (39). Clinical studies have also reported a favorable safety profile for KSS, with no serious adverse events (38). Consistent with this, KSS did not significantly affect the health status of the animals, as indicated by body weight changes, abnormal behavior, or other clinical signs during the 11-day administration period used in this study. Previously, Iba-Tanaka et al. reported that KSS attenuates anxiety-like behavior in PWD rats (18). Consistent with this finding, the present results confirm that KSS decreased anxiety-like behavior in PWD rats, suggesting a potential therapeutic effect of KSS in PMS. As PMS pathogenesis is associated with impaired GABA_A_R response, we performed an *in situ* hybridization assay, noting that KSS administration suppressed the expression of GABA_A_R α4 and δ subunits in the hippocampal CA1 region of PWD rats. KSS consists of 10 crude drugs and contains numerous ingredients. Several constituents in KSS have been reported to have pharmacological activities related to anxiety regulation. For example, geniposide derived from Gardeniae Fructus has been shown to exhibit anxiolytic effects in the social interaction test (40), and paeonol, an ingredient of the crude drug Paeoniae Radix, showed anxiolytic effects in the EPM test when administered to PWD rats (27). In addition, saikosaponin D derived from Bupleuri Radix exhibits estrogen-like effects (41) and hypothalamic-pituitary-adrenal axis regulatory effects (42), whereas isoliquiritigenin derived from Glycyrrhizae Radix has been reported to act as a positive allosteric modulator of GABA_A_Rs (43). These pharmacological properties may collectively contribute, at least in part, to the anxiolytic effects of KSS observed in the present study.

Changes in GABA_A_R subunit expression are associated with fluctuations in neurosteroid levels, such as progesterone and ALLO (14, 44, 45). These neurosteroid-dependent changes in GABAergic signaling influence neuronal excitability and may contribute to behavioral alterations associated with hormonal fluctuations. We tested whether KSS might restore subunit expression by reducing dynamic fluctuations in ALLO levels in PWD rats. Pharmacokinetic analysis showed that plasma progesterone concentrations peaked 30 min after administration, decreasing to less than half within 240 min. Hippocampal progesterone exhibited similar fluctuations to plasma, suggesting rapid brain transfer. ALLO pharmacokinetics were largely similar to those of progesterone. In comparison, ALLO exhibited higher hippocampal/plasma concentration ratios for Cmax and AUC, suggesting that it may readily cross into the brain or undergo enhanced metabolism within it. The plasma progesterone concentration trends in the PWD + KSS group were similar to those in the PWD group. On the other hand, the Cmax of ALLO in the brain tended to be lower in the PWD + KSS group, but not significantly different. Since this study did not collect time-series data from the same animals but sacrificed them at each sampling point, individual variability likely influenced the concentration profiles. Further research is needed for pharmacokinetic analysis. Furthermore, as no pharmacokinetic evaluation of KSS was performed in non-PWD (control) animals, the possibility that KSS may influence neurosteroid levels that could affect GABA_A_R subunit expression cannot be excluded.

Compared to healthy individuals, patients with PMDD exhibit decreased GABA levels during the follicular phase and increased levels during the luteal phase (46), suggesting that changes in GABA concentration may be involved in PMDD symptoms. Thus, we examined changes in the expression of factors involved in GABA production and secretion in the hippocampal CA1 region of PWD rats. In the original comparison, GAT-1 mRNA expression was increased in PWD rats compared with that in control rats (**Supplementary Figure S3**). However, this difference was not maintained after batch-effect adjustment and three-group analysis. Therefore, the present findings do not provide conclusive evidence that PWD robustly alters the expression of genes involved in GABA synthesis, transport, or GABA_A_R α4 subunit regulation in the hippocampal CA1 region. Nevertheless, the observed trend in the original comparison suggests the involvement of altered GABA transport in the pathophysiology of the PWD model and warrants further investigation. To our knowledge, this is the first report on the factors involved in GABA signaling in PWD model animals. Furthermore, we examined the mRNA expression of EGR3 (47), which regulates the transcription of the α4 subunit gene, as a potential reason for the increased GABA_A_R α4 subunit expression. However, no difference in EGR3 mRNA expression was observed between the control and PWD rats. Previous reports on EGR3 mRNA expression targeted the entire hippocampus or dentate gyrus (47, 48). To date, no other study than ours has suggested an association between α4 subunit expression and EGR3 expression in the CA1 region. Further research evaluating EGR3 expression in the CA1 region at multiple time points is necessary to clarify this point. In KSS-treated PWD rats, no effects on GAD1, GAD2, VGAT, GAT-1, or EGR3 mRNA expression were observed. These findings suggest that mechanisms other than GABA or EGR3 production, storage, and transport may be involved in the KSS-induced alteration of GABA_A_R subtype expression. Further research is required to elucidate the detailed mechanism of action of KSS.

GABA_A_R has gained attention as a potential anxiolytic drug target. Benzodiazepine (BDZ) drugs (such as diazepam and alprazolam), which bind to the BDZ binding site of GABA_A_R, enhancing GABA’s effects, are frequently used as anxiolytics and hypnotics. However, due to the high risk of adverse effects such as falls, delirium, dependence, and tolerance, interventions to address long-term and high-dose administration are needed. Chronic BDZ administration reportedly increases α4 subunit expression (49), and BDZ sensitivity decreases after progesterone withdrawal (32), suggesting that increased α4 subunit expression contributes to BDZ tolerance. Since KSS suppressed the increased expression of α4 and δ subunits in PWD rats, its function does not appear to involve binding to GABA_A_R. **Figure 6** shows the proposed mechanism of action of the KSS. Even though KSS-induced changes in subunit expression may offer a way to reverse tolerance caused by BDZ and related drugs, the exact mechanisms behind these changes—and whether KSS truly reverses tolerance—still need to be studied further.

**Figure 6:**
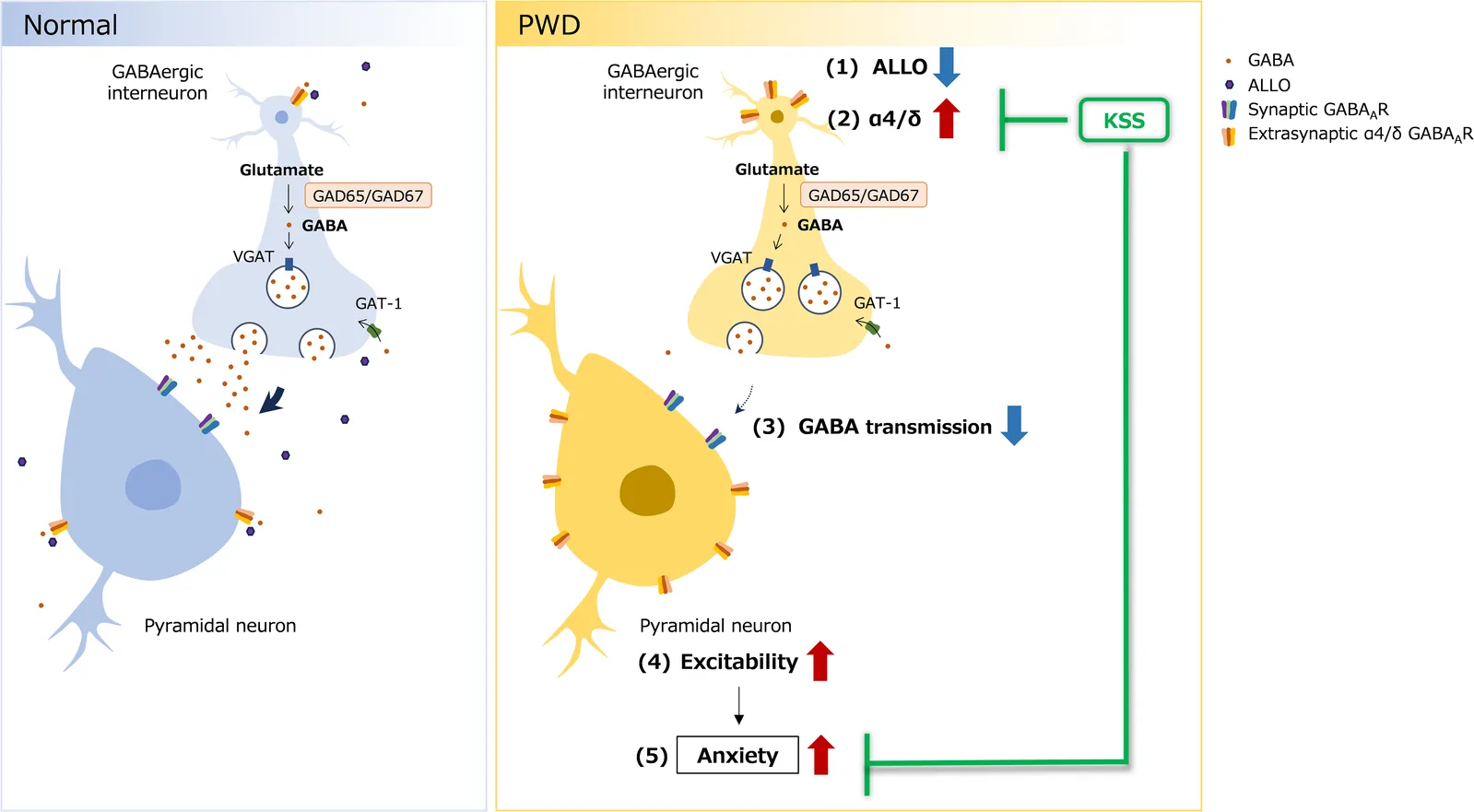
Schematic representation of pathogenesis in the PMS rat model and the pharmacological mechanism underlying KSS’ effects. PWD induces a rapid decrease in ALLO levels (1), which increases GABA_A_R α4 and δ subunit expression (2). These changes may alter GABAergic signaling, resulting in reduced GABA transmission (3), enhanced excitability of pyramidal neurons (4), and anxiety symptoms (5). KSS restores increased GABA_A_R α4 and δ subunit expression, leading to anxiety symptom suppression. PWD, progesterone withdrawal; ALLO, allopregnanolone; GABA_A_R, GABA_A_ receptor; GAD67, glutamate decarboxylase-1 (GAD1/GAD67); GAD65, glutamate decarboxylase-2 (GAD2/GAD65); VGAT, vesicular GABA transporter; GAT-1, GABA transporter 1, KSS, kamishoyosan.

This study has several limitations. First, only a single high KSS dose was examined. The 3% KSS-containing diet corresponded to an estimated exposure of ~2,000 mg/kg/day based on the animals’ mean food intake and body weight. Although this dose was selected to maximize the likelihood of detecting pharmacological effects, using only one dose precluded the assessment of dose–response relationships. Future studies need to include multiple dose levels to more rigorously clarify the exposure–response profile of KSS. Although the EPM test is a well-established and widely used method for assessing anxiety-like behavior in rodents, the use of multiple behavioral paradigms is generally recommended for a more comprehensive evaluation. Thus, the reliance on a single behavioral test should be considered a limitation of the present study. In addition, despite varying observed power across indices (0.42–0.83), effect sizes were consistently large (Hedges’ g = 1.02–1.71). Since the observed (*post hoc*) power is mathematically coupled with the p and provides limited information beyond effect size estimates, our interpretation focused primarily on the latter. Future work should improve generalizability through prospective sample size planning (*a priori* power analysis or sensitivity analysis to determine the smallest detectable effect size for a given sample size). Furthermore, incorporating additional behavioral paradigms in future studies will help validate the anxiolytic-like effects of KSS observed in the present study.

In conclusion, this study demonstrated increased anxiety-like behavior and elevated expression of GABA_A_R α4 and δ subunits in the hippocampal CA1 region in rats after withdrawal from high-dose progesterone. KSS administration decreased anxiety-like behavior in PWD rats through a decrease in GABA_A_R α4 and δ subunit expression in the hippocampal CA1 region. These results suggest that KSS reverses reduced GABA_A_R sensitivity and may be useful for developing future treatments for PMS.

## Data Availability

The original contributions presented in the study are included in the article/**Supplementary Material**, further inquiries can be directed to the corresponding author.
