# Supplementary material for: Kamishoyosan reduces anxiety-like behavior by modulating GABAA receptor subunit expression in a premenstrual syndrome rat model
Source: Front Psychiatry. 2026 Oct 16;17:1799904. doi: 10.3389/fpsyt.2026.1799904 (PMC13720535; doi:10.3389/fpsyt.2026.1799904)
Supplement: Supplementary file 1 [file SupplementaryFile1.docx]

Supplementary Material

# 1 Supplementary Data

## 1.1 Supplementary Table

| Names | Origin | Weight (g) |
| --- | --- | --- |
| Bupleuri Radix | *Bupleurum falcatum* Linné (root) | 3.0 |
| Paeoniae Radix | *Paeonia lactiflora* Pallas (root) | 3.0 |
| Atractylodis Lanceae Rhizoma | *Atractylodes lancea* De Candolle (rhizome) | 3.0 |
| Angelicae Acutilobae Radix | *Angelica acutiloba* Kitagawa (root) | 3.0 |
| Poria | *Wolfiporia cocos* Ryvarden et Gilbertson (sclerotium) | 3.0 |
| Gardeniae Fructus | *Gardenia jasminoides* Ellis (fruit) | 2.0 |
| Moutan Cortex | *Paeonia suffruticosa* Andrews (bark) | 2.0 |
| Glycyrrhizae Radix | *Glycyrrhiza uralensis* Fischer (root and stolon) | 1.5 |
| Zingiberis Rhizoma | *Zingiber officinale* Roscoe (rhizome) | 1.0 |
| Menthae Herba | *Mentha arvensis* Linné var. *piperascens* Malinvaud (terrestrial part) | 1.0 |

**Supplementary Table S1.** Components of kamishoyosan (KSS)

## 1.2 Supplementary Figures

**Supplementary Figure S1.** A three-dimensional high-performance liquid chromatography profile of kamishoyosan, provided by TSUMURA & CO., Tokyo, Japan.

**Supplementary Figure S2.** **Anxiety-like behavior in the elevated plus maze (Batch1 and Batch2)**

(A–H) Behavioral experiments were conducted in two separate batches (batch 1: Control vs. PWD; batch 2: PWD vs. PWD + KSS). Percentage of time spent in the open arms (% open arm time), anxiety index, number of open-arm entries, and percentage of time spent in the enclosed arms (% enclosed arm time) were compared between Control and PWD rats (batch 1, A–D) and between PWD and PWD + KSS rats (batch 2, E–H). Data are presented as the mean ± SEM (n = 7 per group). Statistical analysis was performed using an unpaired *t*-test. Statistically significant differences are indicated as * *p* < 0.05, ** *p* < 0.01.

**Supplementary Figure S3.** **Expression of molecules involved in GABA signaling regulation in the hippocampus**

(A–J) mRNA expression of molecules involved in GABA synthesis, transport, and reuptake as well as regulator of GABAA receptor α4 subunit expression in the hippocampal CA1 region. Control against progesterone withdrawal (PWD) rats (A–E; n = 8) and PWD vs. PWD + kamishoyosan (KSS) rats (F–J; n = 12). Statistical analysis was performed using unpaired *t*-test. Statistically significant differences are indicated as * *p* < 0.05. Relative gene expression levels were calculated within each experimental batch, with the mean expression level of the reference group set to 1.0 (panels A–E, control group; panels F–J, PWD group). GAD1: glutamate decarboxylase-1 (GAD1/GAD67), GAD2: glutamate decarboxylase-2 (GAD2/GAD65), VGAT: vesicular GABA transporter, GAT-1: GABA transporter 1, EGR3: early growth response 3, PWD: progesterone withdrawal, KSS: kamishoyosan.
